# Proliferation of Tau 304–380 Fragment Aggregates through Autocatalytic Secondary Nucleation

**DOI:** 10.1021/acschemneuro.1c00454

**Published:** 2021-11-16

**Authors:** Diana C. Rodriguez Camargo, Eimantas Sileikis, Sean Chia, Emil Axell, Katja Bernfur, Rodrigo L. Cataldi, Samuel I. A. Cohen, Georg Meisl, Johnny Habchi, Tuomas P. J. Knowles, Michele Vendruscolo, Sara Linse

**Affiliations:** † Department of Biochemistry and Structural Biology, Chemical Centre, Lund University, SE-221 00 Lund, Sweden; ‡ Centre for Misfolding Diseases, Department of Chemistry, University of Cambridge, CB2 1EW Cambridge, United Kingdom; § Wren Therapeutics Limited, Clarendon House, Clarendon Road, Cambridge CB2 8FH, United Kingdom; ∥ Cavendish Laboratory, Department of Physics, University of Cambridge, Cambridge CB3 0HE, United Kingdom

**Keywords:** surface catalysis, self-association, precipitation, folding unit, tubulin-associated unit, intracellular aggregation

## Abstract

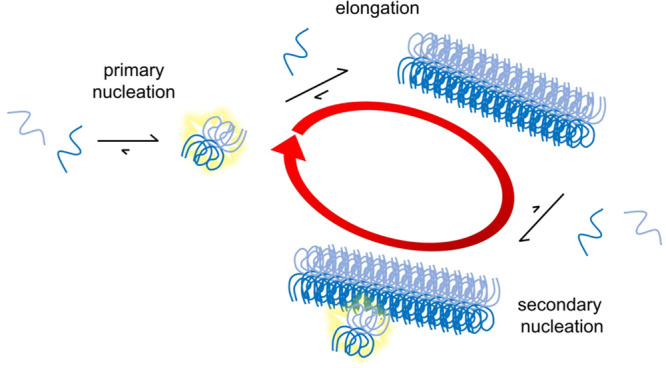

The self-assembly of the protein tau into neurofibrillary tangles is one of the hallmarks of Alzheimer’s disease and related tauopathies. Still, the molecular mechanism of tau aggregation is largely unknown. This problem may be addressed by systematically obtaining reproducible in vitro kinetics measurements under quiescent conditions in the absence of triggering substances. Here, we implement this strategy by developing protocols for obtaining an ultrapure tau fragment (residues 304–380 of tau441) and for performing spontaneous aggregation assays with reproducible kinetics under quiescent conditions. We are thus able to identify the mechanism of fibril formation of the tau 304–380 fragment at physiological pH using fluorescence spectroscopy and mass spectrometry. We find that primary nucleation is slow, and that secondary processes dominate the aggregation process once the initial aggregates are formed. Moreover, our results further show that secondary nucleation of monomers on fibril surfaces dominates over fragmentation of fibrils. Using separate isotopes in monomers and fibrils, through mass spectroscopy measurements, we verify the isotope composition of the intermediate oligomeric species, which reveals that these small aggregates are generated from monomer through secondary nucleation. Our results provide a framework for understanding the processes leading to tau aggregation in disease and for selecting possible tau forms as targets in the development of therapeutic interventions in Alzheimer’s disease.

## Introduction

Alzheimer’s disease (AD) is a devastating neurodegenerative disease of increasing prevalence.^1−3^ The pathology of AD is linked to the self-assembly of the amyloid β (Aβ) peptide into extracellular plaques and of the tubulin-associated unit (tau) protein into intracellular tangles in the brains of AD patients. According to the amyloid cascade hypothesis, extracellular Aβ aggregation leads to intracellular hyper-phosphorylation and aggregation of tau.^4,5^ Significant progress has been made over recent years in understanding the mechanism of Aβ aggregation and its associated toxicity.^6−10^ However, obtaining a comparable understanding of the microscopic mechanism underlying tau self-assembly remains a key challenge.

Tau was isolated and identified in 1975,^11^ and its concentration in neuronal cells is found to be around 2 μM.^12,13^ The protein may misfold and aggregate into neurofibrillary tangles. Such misfolded forms of tau have been identified in several human neurodegenerative diseases, collectively known as tauopathies, including AD, Danish and British dementias, white matter tauopathy with globular glial inclusions, frontotemporal dementia, Pick’s disease, progressive supranuclear palsy (PSP), corticobasal degeneration (CBD), argyrophilic grain disease (AGD), Guam Parkinsonism–dementia complex, tangle-only dementia, Parkinsonism linked to chromosome 17 (FTDP-17T), and Gerstmann–Sträussler–Scheinker disease.^14−17^ Several reports indicate that post-translational modifications may differentiate between these tauopathies.^18−21^ Tau can also be secreted from neurons^22^ and has been observed in blood and cerebrospinal fluid (CSF^23^), in which the levels of phosphorylated tau isoforms serve as biomarkers for AD.^24^ Since aggregated forms of tau have been identified in the extracellular space,^25^ both intracellular and extracellular tau may play a role in the disease. Several reports indicate that misfolded and aggregated forms of tau may be taken up by cells, and tauopathies have been reported to spread by cell-to-cell transmission.^26,27^

**Figure 1:**
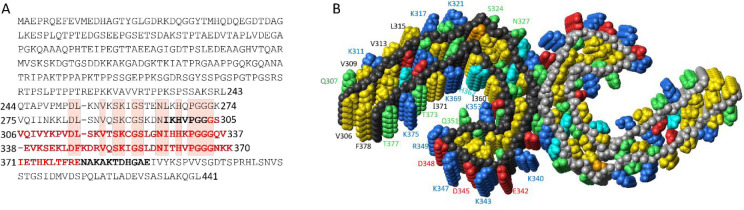
Amino acid sequence of tau441 and structure of tau fibrils from AD patients. (A) Amino acid sequence of tau441 (also called 4R2N) with the four-repeat regions (residues 244–274, 275–305, 306–337, 338–370) aligned and identities shaded. Residues 297–391 are shown in bold; residues 304–380, which represent the tau fragment studied in this work, are shown in red. (B) Five planes of the cryo-EM fibril structure encompassing residues 306–378.^28^ One monomer per plane is shown with a black backbone and one monomer per plane with a gray backbone. The side chains are color-coded with yellow for hydrophobic, red for acidic, blue for basic, cyan for histidine residues, green for other hydrophilic side chains, and orange for Cys322, which was in this work replaced by Ser. The picture was prepared using the file 5O3L.pdb and the program MolMol.^55^

Tau aggregates are composed of β-sheet fibrils that may contain a variety of hyper-phosphorylated isoforms of tau, and several polymorphs have been reported.^19,20,28,29^ In a healthy brain, at least six isoforms of tau, containing between 352 and 441 residues, are produced by alternative splicing of the mRNA from the *MAPT* gene on chromosome 17. Three isoforms contain four microtubule binding repeats (4R) of 31–33 amino acids, and three isoforms contain three such repeats (3R). The sequence of one major isoform (referred to as tau441, 4R2N, or full-length tau) with four repeats is shown in Figure 1A with the residue numbering used in this study. Cryo-electron microscopy (cryo-EM) structures of the so-called paired helical filaments (PHF) extracted from the brains of AD patients revealed that each plane of these PHF fibrils contains two monomers with curved β-strands perpendicular to the fibril axis, and with extended β-sheets formed between identical segments of adjacent planes along the fibril axis^28^ (Figure 1B). The ordered part of each monomer in the fibril comprises residues 306–378, corresponding closely to the shorter of two stable tau peptide fragments (9.5 and 12 kDa) identified in PHFs extracted from AD patient brains.^30^ Tau proteolysis, resulting in a variety of aggregation-prone tau fragments, is known to occur in tauopathies,^31,32^ and recently, a recombinant fragment (residues 297–391) was reported to form highly ordered PHFs, although a detailed mechanistic understanding of its aggregation was not described.^33,34^

Since full-length tau is highly soluble, finding its mechanism of aggregation has remained a major challenge, with previous work using various additives or foreign surfaces to trigger heterogeneous primary nucleation or vigorous agitation to speed up primary and secondary processes. The additives include anionic substances and polymers such as heparin, polyglutamic acids, nucleic acids, negatively charged phospholipids, and surfactants that accelerate fibril formation.^35−42^ Although tau contains two cysteines at positions 291 and 322, the rate of assembly has been found to be inhibited^33^ or independent of cysteine cross-linking;^43,44^ however, an assembly rate was found for wild-type in the presence of reducing agent that was similar to that of a variant with the Cys322 side chain replaced though mutation.^33^

Here, we studied the spontaneous self-assembly of the tau 304–380 fragment into amyloid fibrils under quiescent conditions in pure buffer in sample containers selected for low surface interaction of proteins. The tau 304–380 fragment covers the ordered part of the fibril, that is, residues 306–378 (Figure 1B^28^), plus two extra residues at each terminus, and a Ser residue replaces Cys322 to avoid dimerization of the fragment, which may affect fibril assembly.^33,44^ The terminal residues were added to compensate for the risk of missing structural information at the edge of the core structure in the event that these parts of the fibril were less ordered than the rest of the core. Analogous protocols have previously been successfully applied in the cases of Aβ peptide from AD^6−10^ and α-synuclein from Parkinson’s disease^45,46^ to identify the microscopic steps underlying their self-assembly reaction and are now translated to the study of the tau 304–380 fragment. The formation of amyloid fibrils in supersaturated monomer solutions, without and with the addition of preformed seed fibrils, was followed by the fluorescence of the amyloid-specific dye 4-bis(3-carboxy-4-hydroxyphenylethenyl)-benzene (X34) and validated using cryo-EM. Global kinetics analysis^47^ of data was used to find the minimal combination of microscopic processes that produces a good fit to the experimental data. To distinguish between models and to pinpoint the origin of the small oligomeric intermediates in the aggregation process, which are of relevance for neurotoxicity, we used separate nitrogen isotopes in monomers and fibril seeds.^6^ The results reveal that the aggregation mechanism of the tau fragment is governed by secondary processes and that secondary nucleation on fibril surfaces is the dominant source of new oligomers.

## Results

### Expression and Purification of the Tau Fragment, Tau304–380_C322S

**Figure 2:**
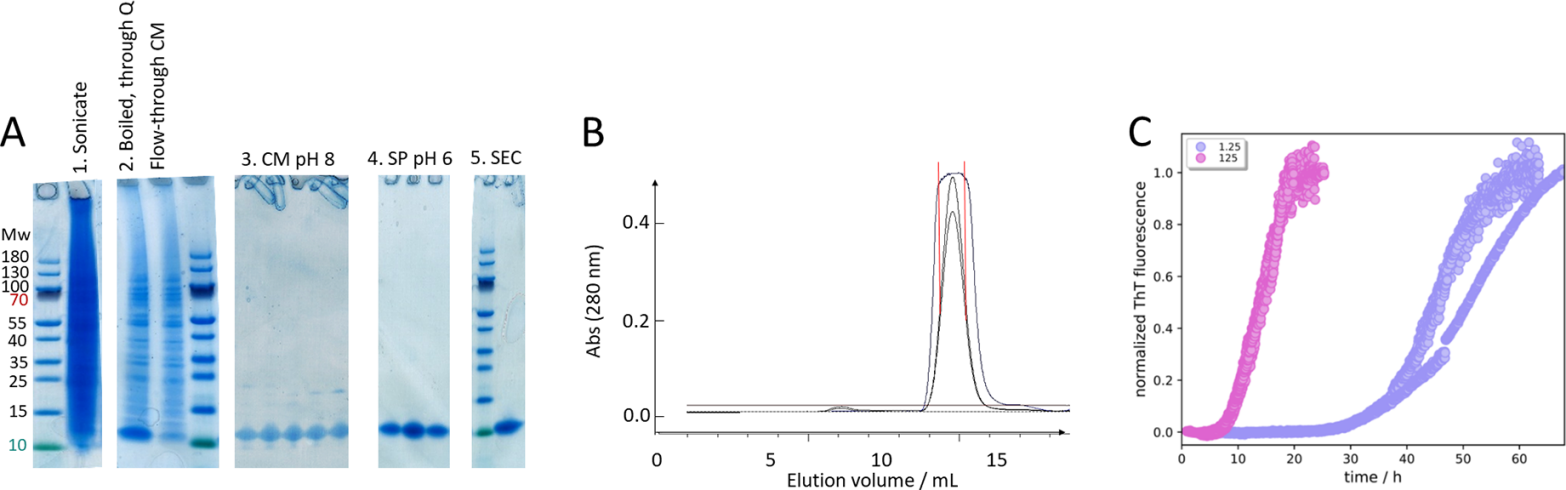
Purification protocol of tau304–380_C322S and quiescent spontaneous aggregation assay. (A) Purification of tau304–380_C322S. The gel lanes show samples after (1) sonication; (2) boiling (1 volume sonicate at 4 °C was poured into 2 volumes boiling buffer at 100 °C, yielding directly 68 °C followed by rapid heating to 95 °C) followed by cooling on ice and passage through Q sepharose at pH 8.0; (3) discarded flow-through of CM Sepharose FF pH 8.0; the *M*_w_ of the standard are given to the left in kDa; (4) pooled IEX elution fractions from CM Sepharose FF pH 8.0; (5) pooled IEX elution fractions from SP Sepharose HP pH 6.0; (6) SEC. The 1st, 5th, and 14th gel lanes show *M*_w_ standard with the green standard protein at 10 kDa. (B) Chromatogram from the SEC isolation of monomer. (C) Aggregation kinetics starting from 1.25 μM (purple) or 125 μM (pink) tau304–380_C322S monomer in 20 mM sodium phosphate, pH 8.0, 0.02% NaN_3_ with 2 μM X34 as a reporter of fibril formation. Data points from three repeats at each concentration are shown.

As a first step toward a mechanistic investigation, we developed methods for the preparation of the tau 304–380 fragment in a highly pure monomeric form. Tau304–380_C322S is prepared with the human sequence with a Cys322 to Ser mutation, with residues numbered as in the human tau 441 isoform (Figure 1A). We expressed this fragment in *Escherichia coli* with no tag except the starting Met residue. This strategy affords a simple and scalable purification protocol based on boiling and two ion exchange steps with intervening switch in pH to alter the elution of the fragment relative to impurities (Figure 2A). The protocol ends with monomer isolation using size-exclusion chromatography (SEC) (Figure 2B), which also exchanges the buffer to the one used in the kinetics experiments. This protocol leads to the isolation of highly pure and monomeric tau304–380_C322S peptide.

### Aggregation Kinetics by X34 Fluorescence

**Figure 3:**
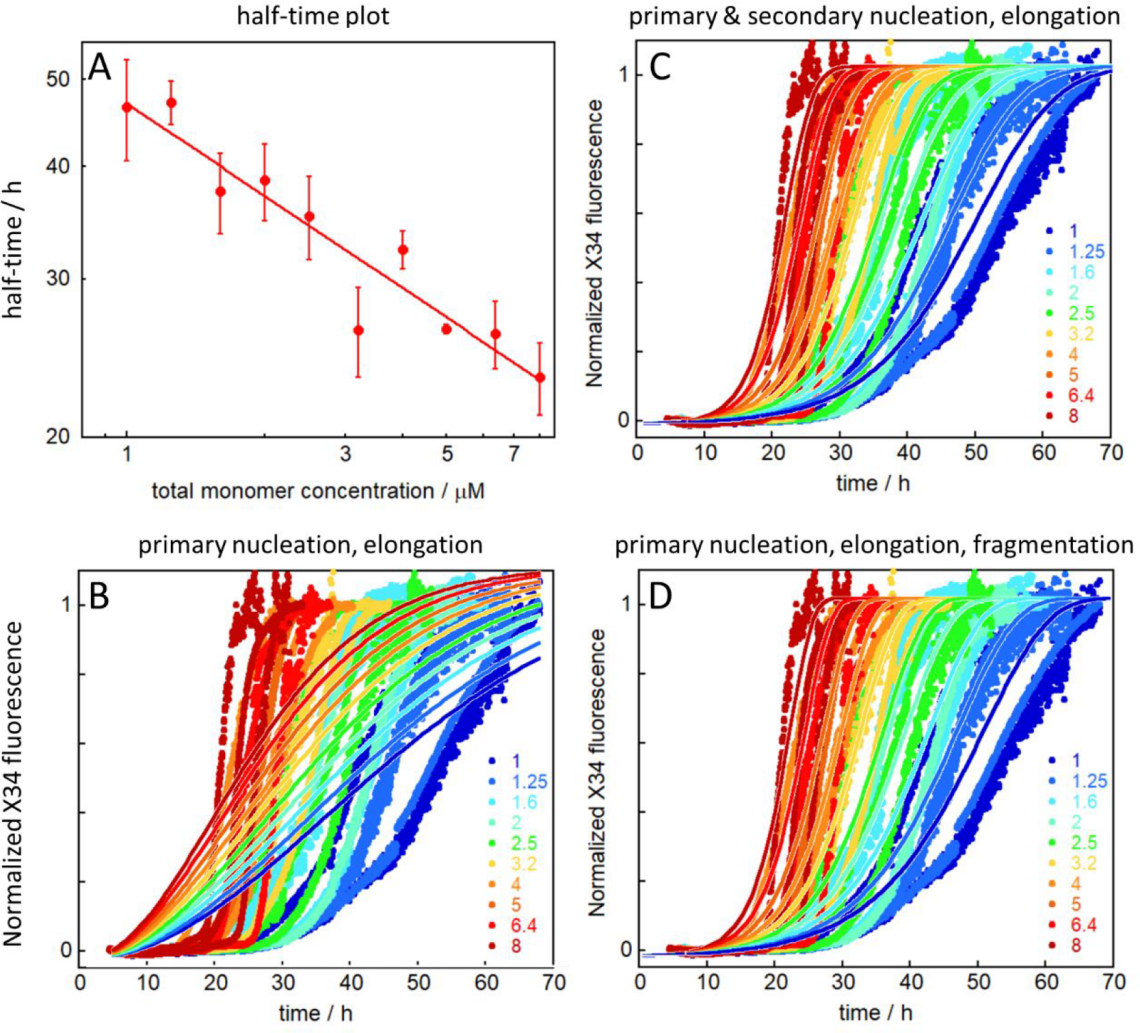
Aggregation kinetics of tau304–380_C322S. Monomer concentrations ranging from 1 to 8 μM were used in 20 mM sodium phosphate, pH 8.0, 0.02% NaN_3_ with 2 μM X34 as a reporter of fibril formation. (A) Half-time of aggregation (*t*_1/2_) as a function of initial monomer concentration with logarithmic axes. The fitted line is a power function with exponent γ = −0.65. (B–D) Normalized kinetics profiles from three repeats at each concentration are shown with the solid lines representing global fits to the data using a model with primary nucleation and elongation (B), a model with primary nucleation, elongation, and multistep secondary nucleation of monomers on fibril surface (C), and a model with primary nucleation, elongation, and fragmentation (D).

As a second step toward a mechanistic investigation, we optimized the conditions for reproducible spontaneous self-assembly of tau304–380_C322S into amyloid fibrils. We measured aggregation kinetics starting from supersaturated solutions of initially monomeric protein at a series of concentrations (in the range 1–200 μM) under quiescent conditions in degassed buffer (20 mM sodium phosphate, 0.2 mM ethylenediaminetetraacetic acid (EDTA), pH 8.0) with 2 μM X34 in wells of low-binding 96-well plates (PEGylated black polystyrene, Coring 3881). The X34 fluorescence intensity was recorded as a reporter of fibril formation at 37 °C, and the X34 concentration was chosen in the low end of the range, giving reproducible and consistent results both at 2.5 and 10 μM peptide (see Supporting Information Figures S1 and S2). Using the ultrapure tau fragment, we were able to observe relatively reproducible aggregation kinetics in the absence of triggers and under quiescent condition, over 2 orders of magnitude in concentration, as exemplified for 1.25 and 125 μM tau304–380_C322S in Figure 2C. All data have a sigmoidal-like appearance with a lag phase, a steep transition, and a final plateau. The data display a long lag time, indicative of low nucleation and/or growth rates; however, there is no evidence of nucleation being slow enough to be stochastic under the studied conditions in bulk solution. Moreover, the kinetics show a clear concentration dependence with faster overall aggregation for higher concentrations of monomer, but with saturation observed above 10 μM (Figure S3). The data in the 1–8 μM range were therefore used in the kinetics analyses, covering the physiological range of tau concentrations.^12,13,48^ The time to reach the reaction midpoint, *t*_1/2_, was extracted from each curve as the point in time at which the X34 fluorescence reached the midpoint between the initial baseline and plateau values. To connect the time scale for each reaction with the corresponding total monomer concentration, the half-time data were fitted by a power function, *t*_1/2_ = *Ac*^γ^, yielding a scaling exponent γ = −0.65 (Figure 3A).

### Kinetics Analysis

Global chemical kinetics analysis of the data was carried out using integrated rate laws describing protein aggregation in terms of the component microscopic steps.^47^ The analysis revealed that models including only primary nucleation and elongation, but not secondary nucleation, do not fit the data (Figure 3B). Strikingly, however, the inclusion of multistep secondary nucleation of monomers on the fibril surface, together with primary nucleation and elongation, resulted in a good global fit (Figure 3C). In this model, secondary nucleation is treated in analogy with the Michaelis–Menten model for enzyme kinetics, with an arrival step for binding of substrate (monomers) to the fibril and a second step involving product formation and release of the formed new fibril nucleus. Accordingly, secondary nucleation saturates at high monomer concentration, and with the reaction order *n*_2_ = 2, the square root of the Michaelis constant, √*K*_M_, corresponds to the monomer concentration at which secondary nucleation is half-saturated. From the fits to the data describing de novo aggregation in the absence of preformed seed material, it is possible to estimate the values of the products of the rate constants for primary nucleation and elongation, *k*_*n*_*k*_*+*_, and for secondary nucleation and elongation, *k*_2_*k*_*+*_, as listed in Table 1. Interestingly, while the data are not well-described by models that lack secondary processes, that is, processes which generate new aggregates at a rate dependent on the concentration of existing aggregates, the data are also reasonably well fitted by a model including primary nucleation, elongation, and the alternative secondary process of fibril fragmentation (Figure 3D). Thus, additional experiments are required to discriminate with certainty the relative roles of monomer-dependent secondary nucleation and fragmentation.

**Table 1:** Fitted Rate Constants^a^

	tau304–380_C322S	Aβ42
*k*_+_*k*_*n*_	<10^–2^ M^–1^ s^–2^^b^	900 M^–2^ s^–2^
*k*_+_*k*_2_	6 × 10^11^ M^–3^ s^–2^	4 × 10^10^ M^–3^ s^–2^
√*K*_M_	30 nM	>6 μM

a The products of rate constants for tau304-380_C322S and in 20 mM sodium phosphate, 0.2 mM EDTA, pH 8.0. Data for Aβ42 from ref (6) are included for comparison.

b Value ill determined; only an upper limit could be determined based on the data. Best fit obtained with n_c_ = 1.

### Seeded Aggregation Kinetics

**Figure 4:**
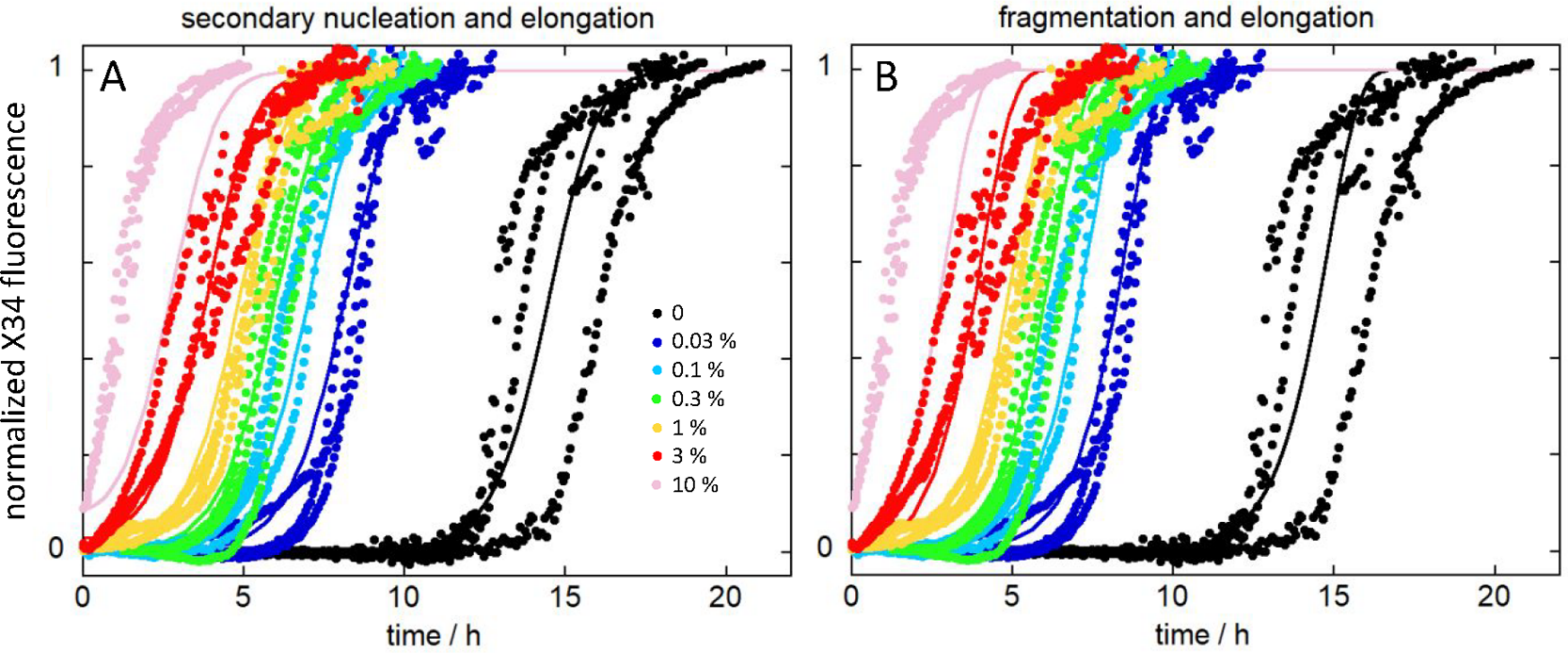
Seeded aggregation kinetics of tau304–380_C322S. We started from freshly isolated monomer alone (black) or supplemented at time zero with preformed seeds (colors). Normalized data at constant monomer concentration and varied seed concentration from 0.03 to 10%. (A,B) Global fits to data using a model of a multistep secondary nucleation and elongation mechanism (A) and a model of fragmentation and elongation (B).

**Figure 5:**
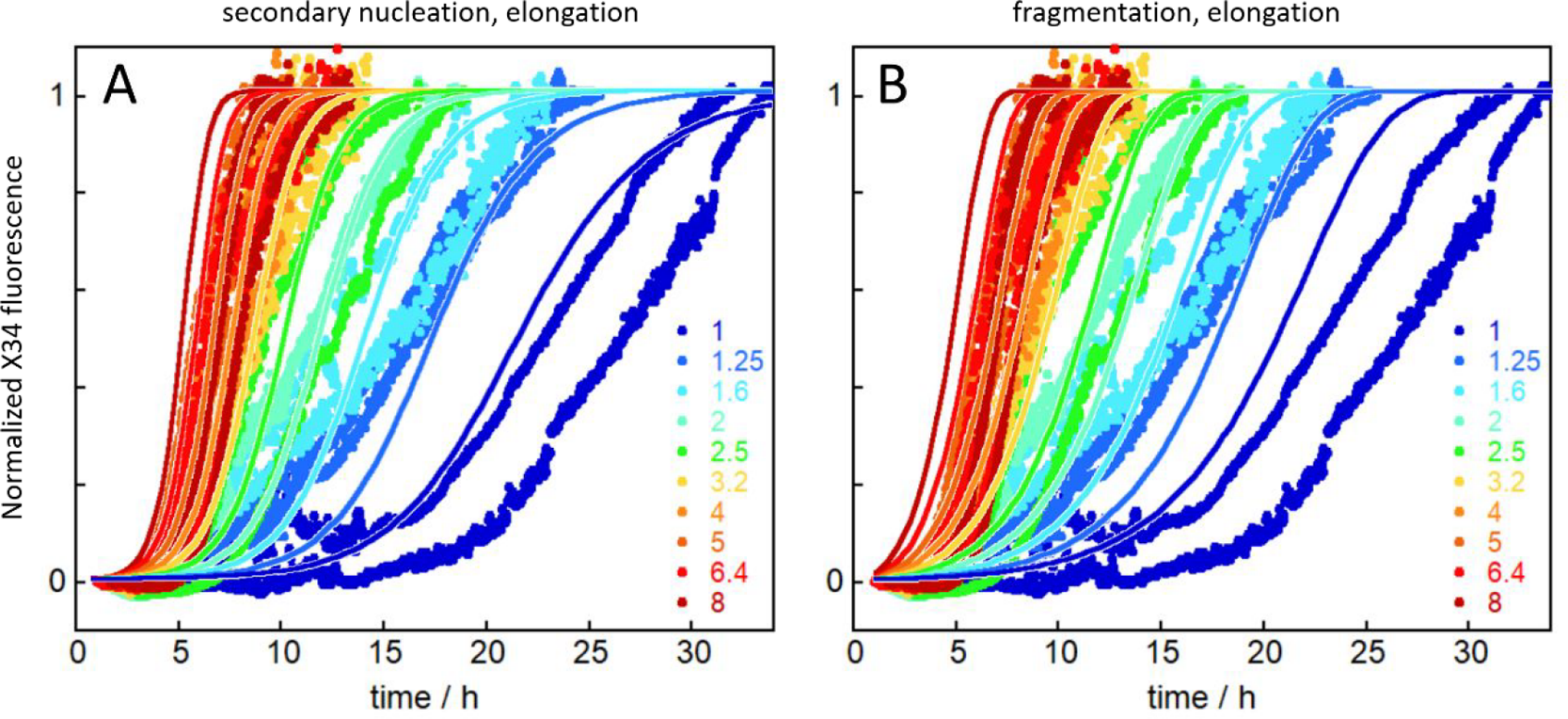
Seeded aggregation kinetics of tau304–380_C322S. We started from freshly isolated monomer supplemented at time zero with preformed seed fibrils. Normalized data are shown at monomer concentrations ranging from 1 to 8 μM, and the initial seed concentration is in each case 0.1% of the monomer concentration. (A,B) Global fits to the data using a model of secondary nucleation and elongation (A) and fragmentation and elongation (B).

Monitoring the aggregation process of proteins in the presence of preformed fibril seeds makes it possible to distinguish secondary processes (secondary nucleation and fragmentation) from primary processes (primary nucleation). Indeed, an observed acceleration of the aggregation process in the presence of added seeds as compared to aggregation in their absence indicates definitively the formation of more aggregates triggered by the fibril seeds.^49^ In the case of tau304–380_C322S in the absence of seeds, the macroscopic aggregation curves as observed using X34 fluorescence are sigmoidal with a lag phase before aggregates can be detected using macroscopic bulk methods. As little as 0.03% seeds (in monomer equivalents) cause a significant shortening of the lag phase, but the sigmoidal shape of the curve is preserved (Figure 4). This observation indicates unambiguously the existence of a secondary, i.e., fibril-dependent, process, the rate of which increases when the amount of fibril increases. As the concentration of seeds is increased, the length of the lag phase is gradually decreased, and at 10% seed, it is completely abolished. Above this concentration, the number of seed ends is high and elongation occurs at a high rate from time zero. Again, the data can be well fitted both by a model dominated by monomer-dependent secondary nucleation and by a fragmentation model. Aggregation as a function of monomer concentration was repeated for a series of samples containing 0.1% seeds (of the respective monomer concentration) at time zero, making the low rate of primary nucleation insignificant. These data can be globally fitted over the entire range from 1 to 200 μM monomer (Figure S4). We show for comparison the data in 1–8 μM concentration range in Figure 5. These data are well-fitted by the model including monomer-dependent secondary nucleation and elongation (Figure 5A) but also reasonably well by the fragmentation model (Figure 5B).

### Origin of Oligomers

**Figure 6:**
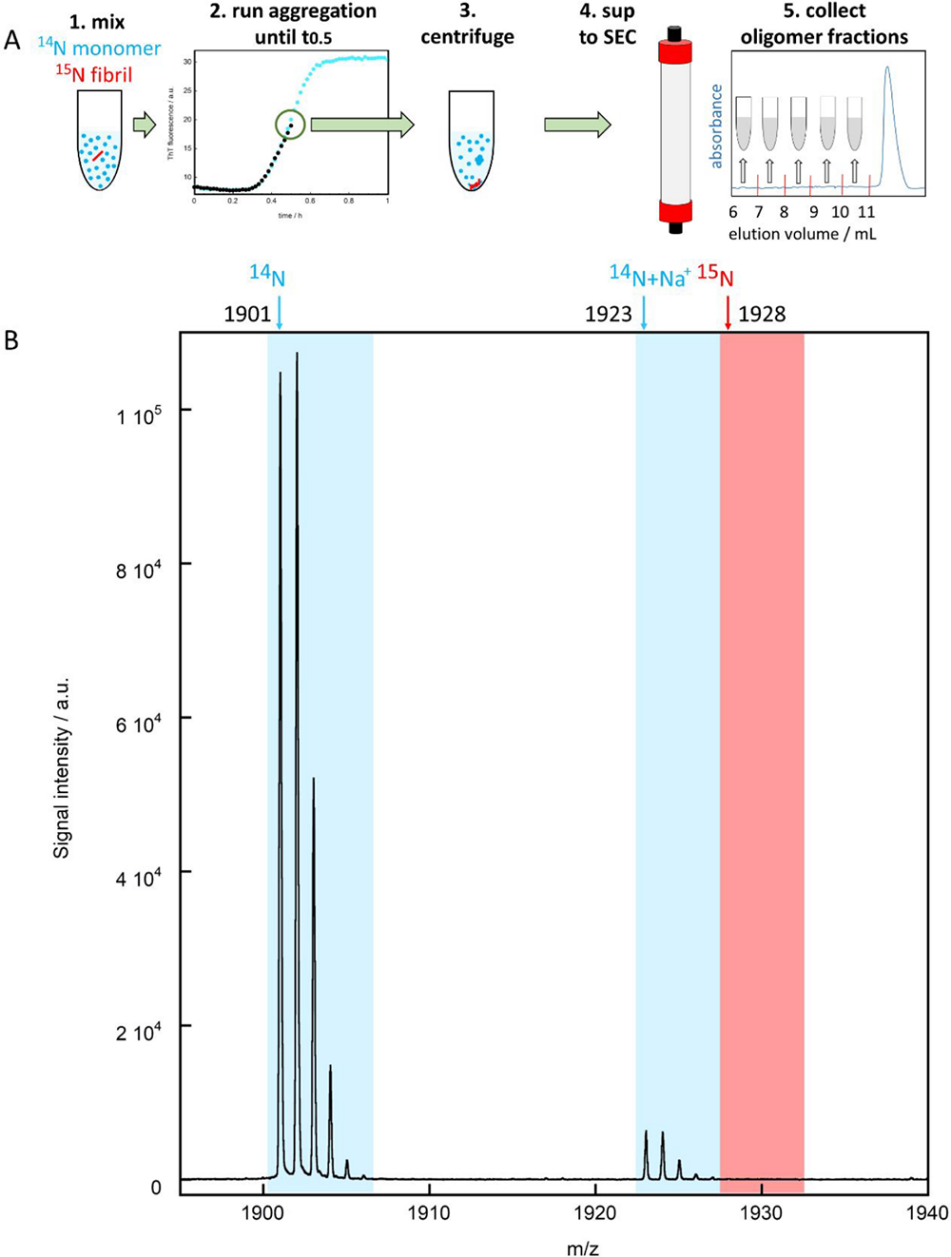
Mass spectrometric analysis of the origin of the oligomers formed by tau304–380_C322S. (A) Outline of the methodology starting from time zero with 10 μM ^14^N monomer supplemented with 0.1 or 1 μM ^15^N seed, sample incubation until *t*_1/2_, sedimentation of fibrils and isolation of oligomers using SEC, followed by tryptic digestion and mass spectrometry. (B) Example of LC-MALDI-TOF-TOF spectrum for the oligomer fraction eluting between 8 and 9 mL for the sample with 1 μM seeds. The blue and red arrows in panel B indicate the location of the monoisotopic peak of ^14^N (1901.00) and ^15^N (1928.00), respectively. Only ^14^N is detected, implying that the collected oligomers created in the seeded reaction originate from the monomer. The peak with monoisotopic mass of 1923.05 is the ^14^N peptide with a sodium (+23-1=+22 Da) adduct. The mass spectra from all spots of fractions 8, 9, and 10 are shown in Figure S3.

The origin of the small oligomeric aggregates appearing as intermediate species during the fibril formation process of tau304–380_C322S was evaluated using mass spectrometry with different isotopes in monomers and seeds in analogy with earlier studies of Aβ42.^6,10,50^ Seeded samples of 10 μM ^14^N monomers with 0.1 or 1.0 μM ^15^N seeds were monitored by X34 fluorescence until *t*_1/2_, centrifuged to remove fibrils, and subjected to SEC to remove monomers and collect oligomer fractions. By following this procedure, we found only tau peptides with ^14^N isotope in the oligomer fractions (Figure 6 and Figure S5). Given the high sensitivity of this assay and by measuring the baseline noise relative to the ^14^N signal, we can conclude that the lack of observable ^15^N signal means that less than 0.035% of the peptides in the oligomeric fraction originate from the original seeds. The initial ^15^N seeds make up 100% of the total aggregate mass at time zero but, at the half-time, only ∼17% of the total aggregate mass due to the formation of new fibrils from ^14^N monomer. Thus, even in a pure fragmentation system, the proportion of ^15^N peptide from initial seeds is expected to be less than 17% in small aggregates. However, the failure to observe any ^15^N above the 0.035% detection limit in the oligomer fraction thus strongly suggests that monomers are the main source of formation of small aggregates in a seeded sample, ruling out the possibility that they are generated through fragmentation of existing fibrils. We thus conclude that the predominant source of oligomers is secondary nucleation of monomers in a reaction catalyzed by fibrils.

### Fibril Morphology by Cryo-EM

**Figure 7:**
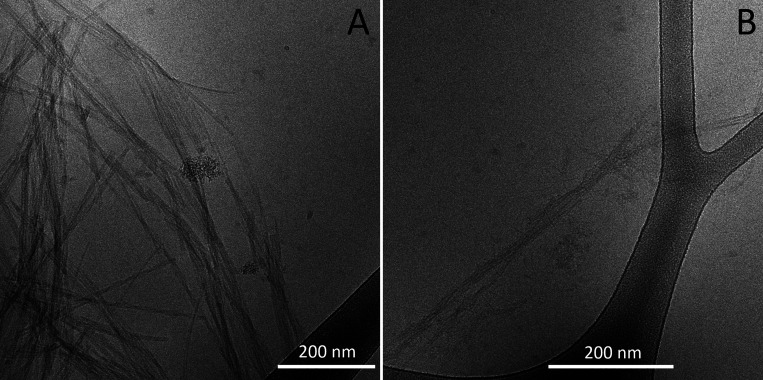
Cryo-EM images of tau304–380_C322S amyloid fibrils. (A,B) Samples were analyzed after reaching the plateau in X34 fluorescence (A) and at *t*_1/2_ of the kinetics run (B). Additional frames are shown in Figures S6 and S7.

Cryo-EM was used to provide information on the morphology of the aggregates formed by the tau fragment. Samples of 25 or 200 μM tau304–380_C322S were monitored by X34 fluorescence, collected directly after reaching the plateau, and found to contain long fibrils with a tendency to associate laterally (Figure 7A). Another sample of 200 μM tau304–380_C322S was taken for imaging at *t*_1/2_, showing laterally associated long fibrils and in their vicinity a number of shorter filaments (Figure 7B), which may be the results of the monomer-dependent secondary nucleation process detected above.

## Discussion

Previous studies of tau aggregation have included various additives to trigger heterogeneous primary nucleation or have used agitation to accelerate primary and secondary processes. Such approaches have been based on observations that tau does not readily form fibrils spontaneously under quiescent and physiological conditions in terms of pH, ionic strength, temperature, and concentration.^35−44^ Here, we have investigated whether a highly pure fragment of tau is able to spontaneously aggregate in the absence of triggers.

A key challenge in elucidating the microscopic mechanisms underlying tau aggregation has been the generation of reproducible experimental data. Here, we have used macroscopic samples at concentrations expected to be outside the stochastic regime (a 100 μL sample of 5 μM protein corresponds to 3 × 10^14^ molecules per sample) and thus behave in a reproducible manner, given high sample purity and careful control of other experimental variables. Indeed, we have first established a reliable protocol for isolation of highly pure monomeric tau fragment comprising residues 304–380 (Figure 2). Thereafter, we optimized the experimental conditions to allow for reproducible observation of the spontaneous self-assembly of this fragment into amyloid fibrils under quiescent conditions. Supersaturated solutions of initially monomeric tau fragments at a series of concentrations were created by a temperature jump from 0 °C (high solubility) to 37 °C (lower solubility), and the formation of amyloid fibrils was followed by X34 fluorescence, with the formation of fibrillar aggregates validated using cryo-EM.

Global kinetics analysis^47^ of the de novo aggregation data reveals that a model including primary nucleation and elongation alone is not sufficient to reproduce the observed data. However, two minimal models including secondary processes produce a good fit to the experimental data. The model including secondary nucleation of monomers on the fibril surfaces is only marginally better (5% lower error square sum) than the model including fragmentation of fibrils. Thus, these data provide no discrimination between these two models. The data from experiments performed for solutions of monomers supplemented with known concentrations of preformed fibrils (Figures 3 and 4) provide only slightly better discrimination in favor of monomer-dependent secondary nucleation.

To further discriminate the two potential classes secondary processes, we used separate isotopes in monomers (^14^N) and seeds (^15^N) to pinpoint the origin of the oligomers. This approach identifies monomers from solution as the main component of the oligomers, therefore indicating that the dominant process responsible for the generation of oligomers is the secondary nucleation of monomers on fibril surfaces, rather than the fragmentation of existing fibrils.

We note that approaches similar to the one described here may be suitable for establishing the mechanism of aggregation of other tau forms, including the six full-length isoforms and their post-translationally modified variants. However, one may expect the solubility to be considerably higher and the lag time considerably longer for these other species. A recent combined Monte Carlo simulation and experimental study showed that extending an amyloidogenic peptide with a non-amyloidogenic segment of equal length leads to about 20-fold longer lag time.^51^ Based on these results, the 441-residue full-length isoform could be expected to aggregate on 3 orders of magnitude longer time scale than the 77-residue amyloid core fragment studied here. We anticipate that seeded aggregation kinetics will be instrumental for bringing the reaction of the longer isoforms into a reasonable experimental time frame.

**Figure 8:**
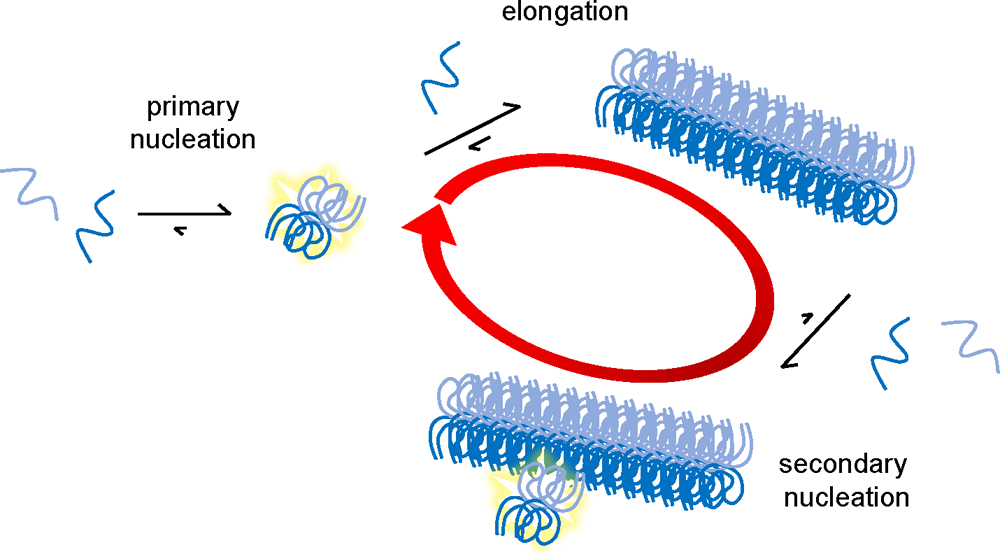
Aggregation model for the tau fragment. The model includes three classes of microscopic steps: primary nucleation (very slow), elongation by monomer addition, and secondary nucleation of monomers on the fibril surface and is compatible with all the data collected in the present work. The red circular arrow indicates the autocatalytic feedback loop consisting of secondary nucleation and elongation, and the faint yellow stars indicate nuclei.

We also note that fibrils formed from truncated forms of tau may act in vivo as seeds for the aggregation of full-length tau, and that it is also possible that cellular factors may induce heterogeneous primary nucleation of tau in the cellular environment. In any case, our results indicate that the proliferation of tau aggregates can take place by fibril-dependent secondary processes, which, being autocatalytic, may proceed very rapidly (Figure 8). This aspect is particularly relevant in the spreading of tau aggregation across different regions of the brain,^52^ as the ability of the traveling seeds to proliferate may influence the rate of the overall process.

It might also be of relevance to compare the overall rate as well as the rate constants for the microscopic processes to other amyloid systems. Such comparison is most straightforward under identical experimental conditions. In Table 1, we show the estimated rate constant products for the tau fragment alongside values for Aβ42 under identical solution conditions (20 mM sodium phosphate, 0.2 mM EDTA, pH 8.0, 37 °C), mechanical (quiescent) and surface conditions (PEGylated half-area 96-well plates). The rate constant products for secondary pathways are much more similar (within a factor of 15) than for primary pathways (differing by at least 5 orders of magnitude, although precise determination is challenging due to the low rate for the tau fragment). These results indicate that a key difference between the two peptides lies in the primary nucleation rate, which is much slower for the tau fragment than for Aβ42.

In summary, we have found that the aggregation mechanism of the tau 304–380 fragment is governed by a secondary process, and that the generation of oligomers is dominated by the secondary nucleation of monomers on fibril surfaces. The discovery that the mechanism of proliferation of aggregates is similar for tau and Aβ suggests that drugs targeting tau may be developed with a similar mechanism of action of those that currently target Aβ, including the recently approved aducanumab, which inhibits Aβ oligomer formation by targeting secondary nucleation.^50^

## Materials and Methods

### Chemicals

All chemicals were of analytical grade. 1,4-Bis(3-carboxy-4-hydroxyphenylethenyl)benzene, X34, was synthesized in-house. Reagents used in this synthesis were purchased from Sigma-Aldrich, Germany, and X-34 was synthesized using the method previously described.^53,54^ Tetraethyl *p*-xylylenediphosphonate was first dissolved in anhydrous dimethylformamide (DMF). Dry potassium butoxide was then added. 5-Formylsalicylic acid dissolved in DMF was carefully added to the resulting mixture and stirred overnight. The reaction mixture was then added to distilled water to precipitate the desired product and filtered. Finally, the powder was washed with ethyl acetate to remove any remaining organic reagents, and the powder was dried overnight under vacuum, yielding a bright yellow-green X34.

### Expression of Tau304–380_C322S

A synthetic gene with *E. coli* optimized codons for the fragment tau304–380_C322S (residue numbering referring to human tau 441) was cloned between NdeI and *Bam*HI in a Pet3a plasmid (purchased from Genscript, Piscataway, New Jersey) to code for the fragment “as is” (MGSVQIVYKPVDLSKVTSKSGSLGNIHHKPGGGQVEVKSEKLDFKDRVQSKIGSLDNITHVPGGGNKKIETHKLTFRE) with no tags except the starting Met, and with Cys322 replaced by Ser. The fragment was expressed in *E. coli* BL21 DE3 PlysS star in overnight express medium (2.5 mM Na_2_HPO_4_, 2.5 mM KH_2_PO_4_, 12 mM (NH_4_)_2_SO_4_, 1 mM MgSO_4_, 0.1 g/L glucose, 0.4 g/L lactose, 1 g/L glycerol, 10 g/L NaCl, 10 g/L tryptone, 5 g/L Bacto yeast extract, 50 mg/L ampicillin, 30 mg/L chloramphenicol). ^15^N-labeled tau304–380_C322S was expressed in M9 minimal medium with ^15^NH_4_Cl as the sole nitrogen source and using isopropyl β-d-1-thiogalactopyranoside for the induction of protein expression.

### Purification of Tau304–380_C322S

The purification protocol is based on sonication, boiling, ion exchange, and SEC. The pellet from a 2 L culture was sonicated in 50 mL of ice-cold 10 mM Tris/HCl, 1 mM EDTA, pH 8.0 (buffer A) for 2 min (50% duty cycle −1 on 1 s off) and centrifuged at 18,000*g* for 10 min. The supernatant (4 °C) was poured into 100 mL of boiling buffer A (100 °C) under stirring in a glass beaker, thus immediately raising the temperature to 69 °C, followed by rapid heating to 95 °C. The solution was cooled by placing the beaker in ice and swirling the solution, followed by centrifugation at 18,000*g* for 10 min to pellet the precipitated *E. coli* proteins. The supernatant was passed through a 20 mL Q Sepharose resin equilibrated in buffer A and then applied to a 20 mL CM Sepharose fast flow column (GE Healthcare, earlier batches) or a 20 mL SP Sepharose high-performance column (GE Healthcare, later batches) equilibrated in buffer A. The column was washed with 50 mL of buffer A and eluted with a linear salt gradient from 0 to 300 mM NaCl in buffer A. The elution of tau304–380_C322S was monitored by the absorbance at 214, 260, and 280 nm, and by SDS PAGE. The peak fractions were pooled, diluted with an equal volume of 20 mM MES pH 5.6, and the pH was adjusted to 6.0. This solution was applied to a 20 mL of SP Sepharose high-performance column equilibrated in 20 mM MES pH 6.0 (buffer B). The column was washed with 50 mL of buffer B and eluted using a linear salt gradient from 50 to 500 mM NaCl in buffer B. The elution of tau304–380_C322S was monitored by the absorbance at 214, 260, and 280 nm and by SDS-PAGE. The peak fractions were pooled, lyophylized, dissolved in 6 M GuHCL, pH 8.0, and further purified using size-exclusion chromatography on a 26 × 600 mm Superdex75 column equilibrated in 20 mM sodium phosphate, 0.2 mM EDTA, pH 8.0. The elution of tau304–380_C322S was monitored by the absorbance at 214, 260, and 280 nm and by SDS-PAGE. The monomer fractions were pooled and lyophylized as multiple identical aliquots.

### Aggregation Kinetics

An aliquot of purified and lyophylized monomeric tau304–380_C322S was dissolved in 1 mL of 6 M GuHCl, pH 8.0, and monomers were again isolated using SEC on a Superdex 75 10/300 column (GE Healthcare) operating in degassed 20 mM sodium phosphate, 0.2 mM EDTA, pH 8.0. The central part of the monomer peak was collected in low binding tube (Axygen) in an ice box, supplemented with 2 μM X34, and diluted to provide a series of samples with concentrations ranging from 0.8 to 250 μM in degassed buffer (20 mM sodium phosphate, 0.2 mM EDTA, pH 8.0, 2 μM X34) in low binding tubes (Axygen) in an ice box. The solutions were pipetted into wells of low-binding 96-well plates (PEGylated black polystyrene, Coring 3881), which was placed in a plate reader equilibrated at 37 °C. Such a temperature jump creates supersaturated tau304–380_C322S solutions, and the aggregation of these solutions was followed under quiescent condition at 37 °C by monitoring the X34 fluorescence intensity using excitation filter of 355 or 380 nm and emission filer of 460 nm, through the bottom of the plate in a Fluostar Omega or Optima plate reader (BMG). The experiments were performed at relatively low X34 concentration (2 μM), based on initial tests showing acceleration of tau aggregation in the presence of thioflavin S as well in the presence of X34 at concentrations above 20 μM, while thioflavin T was discarded because this dye gave relatively low fluorescence signal in the presence of the fibrils of this tau fragment.

### Cryo-EM

Samples of 25 or 200 μM tau304–380_C322S were monitored by X34 fluorescence and collected directly after reaching the plateau. One sample of 200 μM tau304–380_C322S was taken for imaging at *t*_1/2_. Another sample was imaged after being stored for 1 month. Specimens for electron microscopy were prepared in a controlled environment vitrification system to ensure stable temperature and to avoid loss of solution during sample preparation. The specimens were prepared as thin liquid films, <300 nm thick, on lacey carbon film copper grids and plunged into liquid ethane at −180 °C. This leads to vitrified specimens, avoiding component segmentation and rearrangement, and water crystallization, thereby preserving original microstructures. The vitrified specimens were stored under liquid nitrogen until measured. A Fischione model 2550 cryo-transfer tomography holder was used to transfer the specimen into the electron microscope, JEM 2200FS, equipped with an in-column energy filter (Omega filter), which allows zero-loss imaging. The acceleration voltage was 200 kV, and zero-loss images were recorded digitally with a TVIPS F416 camera using SerialEM under low-dose conditions with a 30 eV energy selecting slit in place. Experiments were also conducted using another electron microscope. Here, samples stored under liquid nitrogen were transferred using an Oxford CT3500 cryoholder and its workstation into the electron microscope, Philips CM120 Biotwin Cryo, equipped with a postcolumn energy filter, Gatan GIF100. The acceleration voltage was 120 kV, and images were recorded digitally with a CCD camera under low electron dose conditions.

### Origin of Oligomers

Mass spectrometry with isotope discrimination was used to identify the origin of oligomers. ^15^N-Labeled monomers were isolated and allowed to form fibril seeds. These seeds were added to final concentrations of 0.1 (1%) and 1.0 μM (10%), respectively, to freshly purified 10 μM ^14^N-labeled monomers, each sample (1.2 mL) split into 12 wells of a 96-well plate, and the aggregation process monitored using X34 fluorescence until reaching *t*_1/2_. Each sample was then collected and centrifuged for 2 min at 31,000*g*, and the upper 1 mL of the supernatant loaded onto a Superdex75 column operated in 20 mM ammonium acetate pH 8.0 and 1 mL fractions eluting before the monomer were collected. This procedure thus removes fibrils and monomers, while oligomers are isolated for quantification. Each gel filtration fraction was lyophilized, dissolved in 10 μL of water, and subjected to digestion with trypsin protease overnight. The samples were separated on a nano-LC system (Dionex, Thermo Fisher, CA, USA) with a C18 reversed phase pre- and separation column and spotted together with α-cyano-4-hydroxycinnamic acid matrix solution on a stainless steel MALDI sample plate, prior to the mass spectrometric analysis in positive reflector mode using an Autoflex Speed MALDI time-of-flight (TOF)/TOF mass spectrometer (Bruker Daltonics, Bremen, Germany). The peptide used for analysis corresponds to residues 322–340: SGSLGNIHHKPGGGQVEVK, 27 nitrogen atoms, MH^+^(^14^N) = 1901.00 Da, MH^+^(^15^N) = 1928.00 Da.
