# Supplementary material for: Proliferation of Tau 304–380 Fragment Aggregates through Autocatalytic Secondary Nucleation
Source: ACS Chem Neurosci. 2021 Nov 16;12(23):4406–15. doi: 10.1021/acschemneuro.1c00454 (PMC8640994; doi:10.1021/acschemneuro.1c00454)
Supplement: Supplementary file 1 — cn1c00454_si_001.pdf [file cn1c00454_si_001.pdf]

Supplementary Figures

Proliferation of tau AD fragment aggregates through autocatalytic secondary nucleation

Diana C. Rodriguez Camargo[1,3*], Eimantas Sileikis[3], Sean Chia[3], Emil Axell[1], Katja Bernfur[1], Rodrigo L Cataldi[2], Samuel Cohen[3], Georg Meisl[2], Johnny Habchi[3], Tuomas PJ Knowles[2,4], Michele Vendruscolo[2], Sara Linse[1*]

1. Department of Biochemistry and Structural Biology, Chemical Centre, Lund University, SE-221 00 Lund, Sweden
2. Centre for Misfolding Diseases, Department of Chemistry, University of Cambridge, CB2 1EW Cambridge, UK
3. Wren Therapeutics Limited, Clarendon House, Clarendon Road, Cambridge CB2 8FH, UK.
4. Cavendish Laboratory, Department of Physics, University of Cambridge, Cambridge, CB3 0HE, UK

* Corresponding author

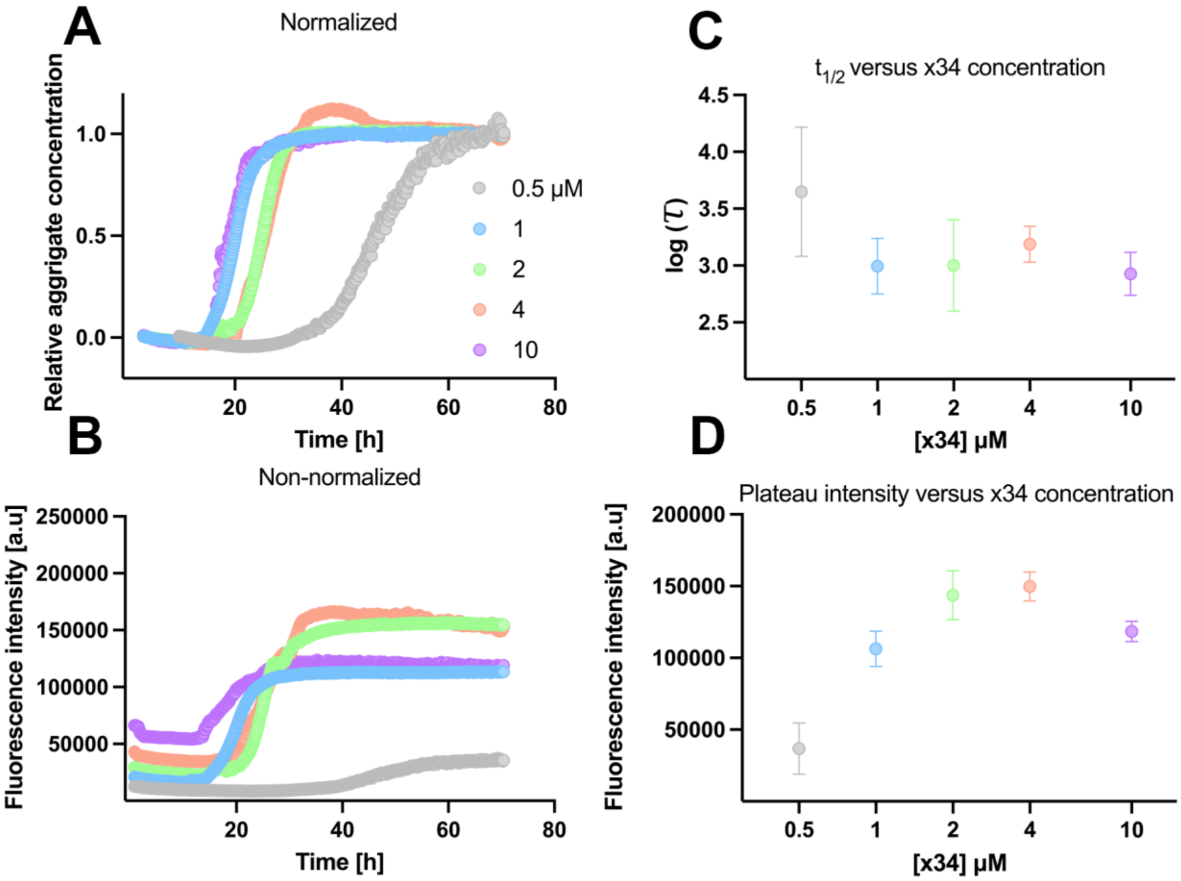

Figure S1. The effect of X34 concentration on signal intensity and aggregation kinetics was investigated for reactions starting from 2.5 µM tau304-380_C322S monomer with x34 concentrations ranging from 0.5-10 µM. (**A**) Normalized and (**B**) non-normalized fluorescence intensity versus time. The median trace of five is shown at each X34 concentration. (**C**) Mean aggregation half-time (τ) and standard deviation over 5 replicates at each X34 concentration. (**D**) Mean final plateau fluorescence intensity and standard deviation over 5 replicates at each X34 concentration.

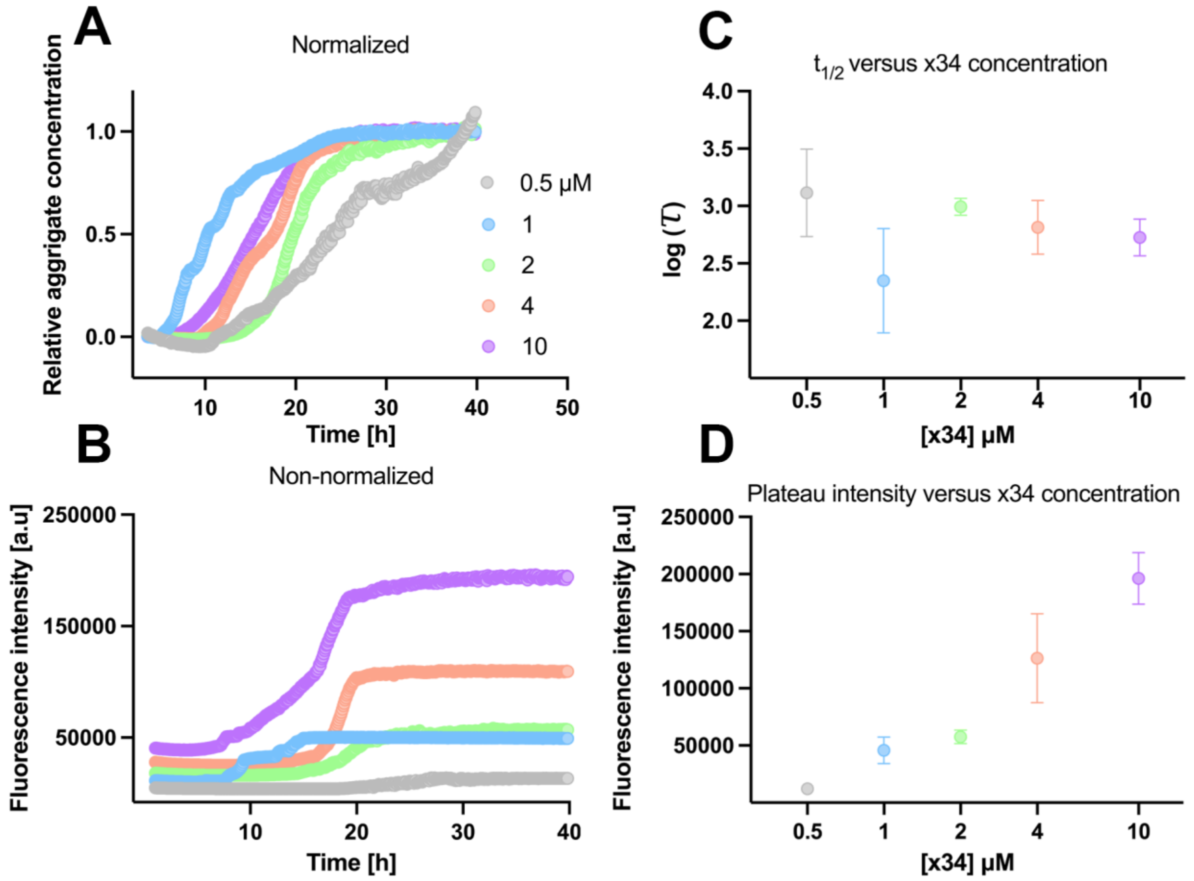

Figure S2. The effect of X34 concentration on signal intensity and aggregation kinetics was investigated for reactions starting from 10 μM tau304-380_C322S monomer with X34 concentrations ranging from 0.5-10 μM. (**A**) Normalized and (**B**) non-normalized fluorescence intensity versus time. The median trace of five is shown at each X34 concentration. (**C**) Mean aggregation half-time (τ) and standard deviation over 5 replicates at each X34 concentration. (**D**) Mean final plateau fluorescence intensity and standard deviation over 5 replicates at each X34 concentration.

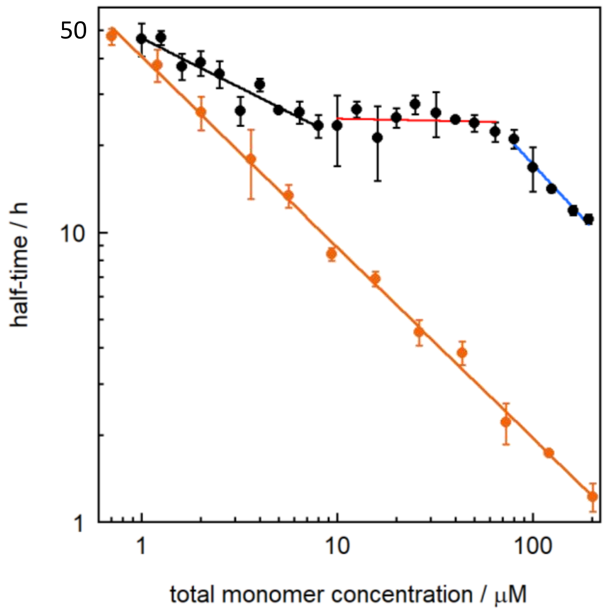

Figure S3. Concentration dependence of the half-time of aggregation for tau tau304-380_C322S. Experiments were carried out with 1 to 200 μM monomer in 20 mM sodium phosphate, pH 8.0, 0.02% NaN_3 with 2 μM X34 as a reporter of fibril formation. Half-time of aggregation ($t_{1/2}$) as a function of initial monomer concentration with logarithmic axes. A) Data (average and standard deviation over three repeats) for reactions starting from pure monomer are shown in black, and data for reactions starting from monomer supplemented with 0.1% seed in monomer units is shown in orange. The data obtained without seeds seem to fall into three regimes, and because of the saturation effects seen above 10 μM, data from the regime 1-8 μM was used in the kinetic analyses (Fig. 3). Data from the same or full range were used in kinetic analysis of the data for seeded samples.

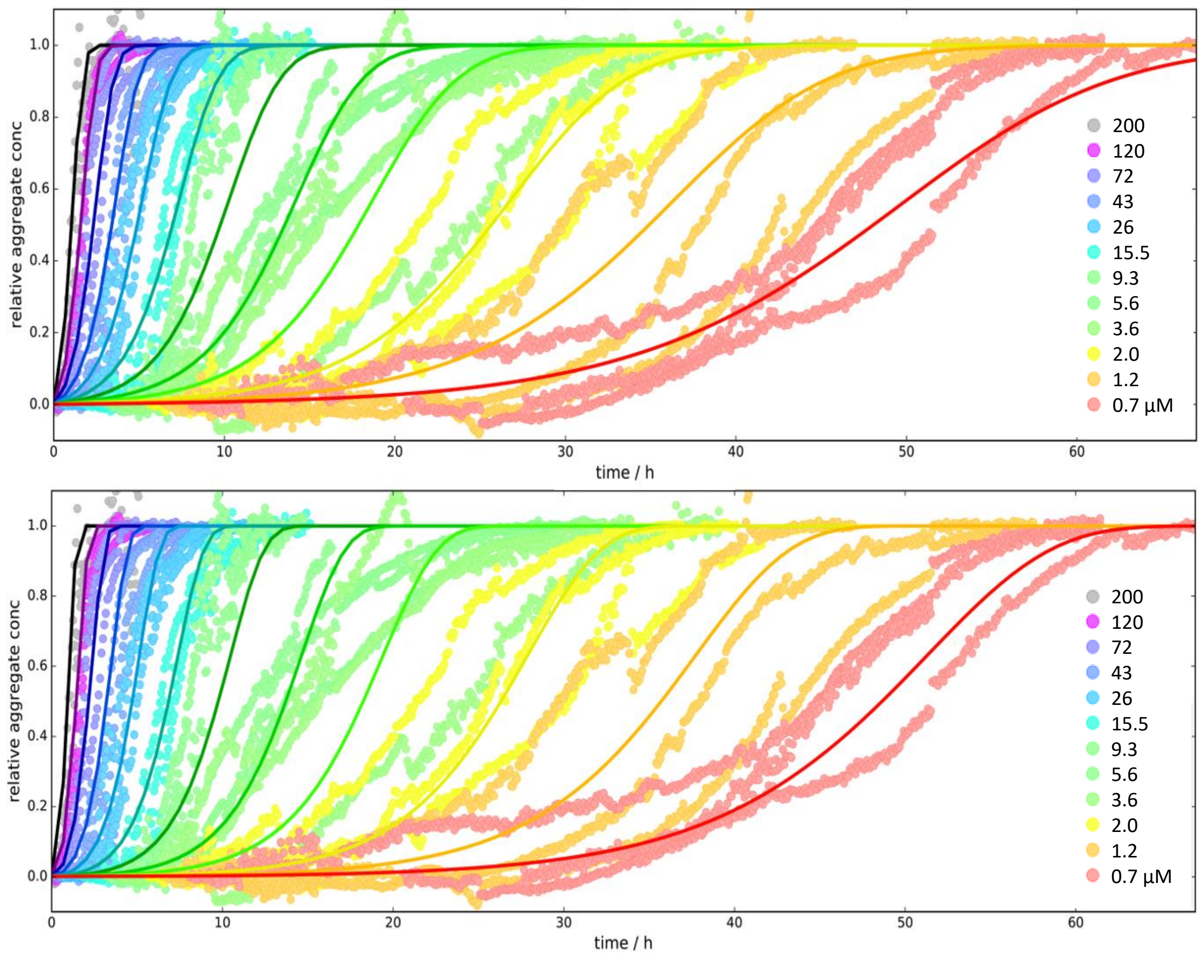

Figure S4. Seeded aggregation kinetics of tau304-380_C322S. Freshly isolated monomer was supplemented at time zero with 0.1% pre-formed seeds. Normalized data at monomer concentrations ranging from 1-200 µM and the initial seed concentration is in each case 0.1% of the monomer concentration. (**A)** Best fit allowing for secondary nucleation and elongation. (**B)** Best fit allowing for fragmentation and elongation.

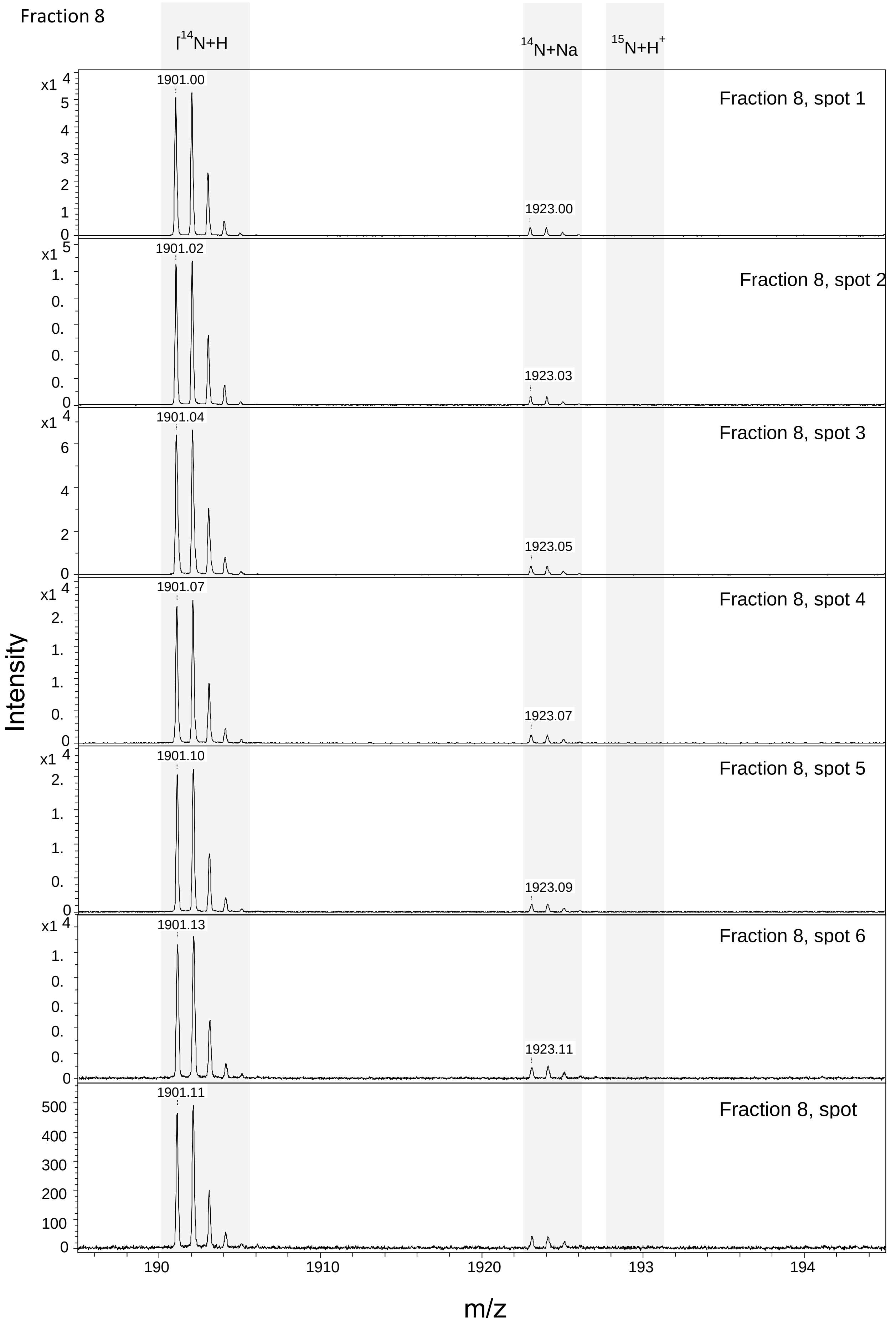
Fraction 8
[14]N+H
[14]N+Na
[15]N+H+
Fraction 8, spot 1
1901.00
1923.00
Fraction 8, spot 2
1901.02
1923.03
Fraction 8, spot 3
1901.04
1923.05
Fraction 8, spot 4
1901.07
1923.07
Fraction 8, spot 5
1901.10
1923.09
Fraction 8, spot 6
1901.13
1923.11
Fraction 8, spot
1901.11
Intensity
m/z
190
1910
1920
193
194

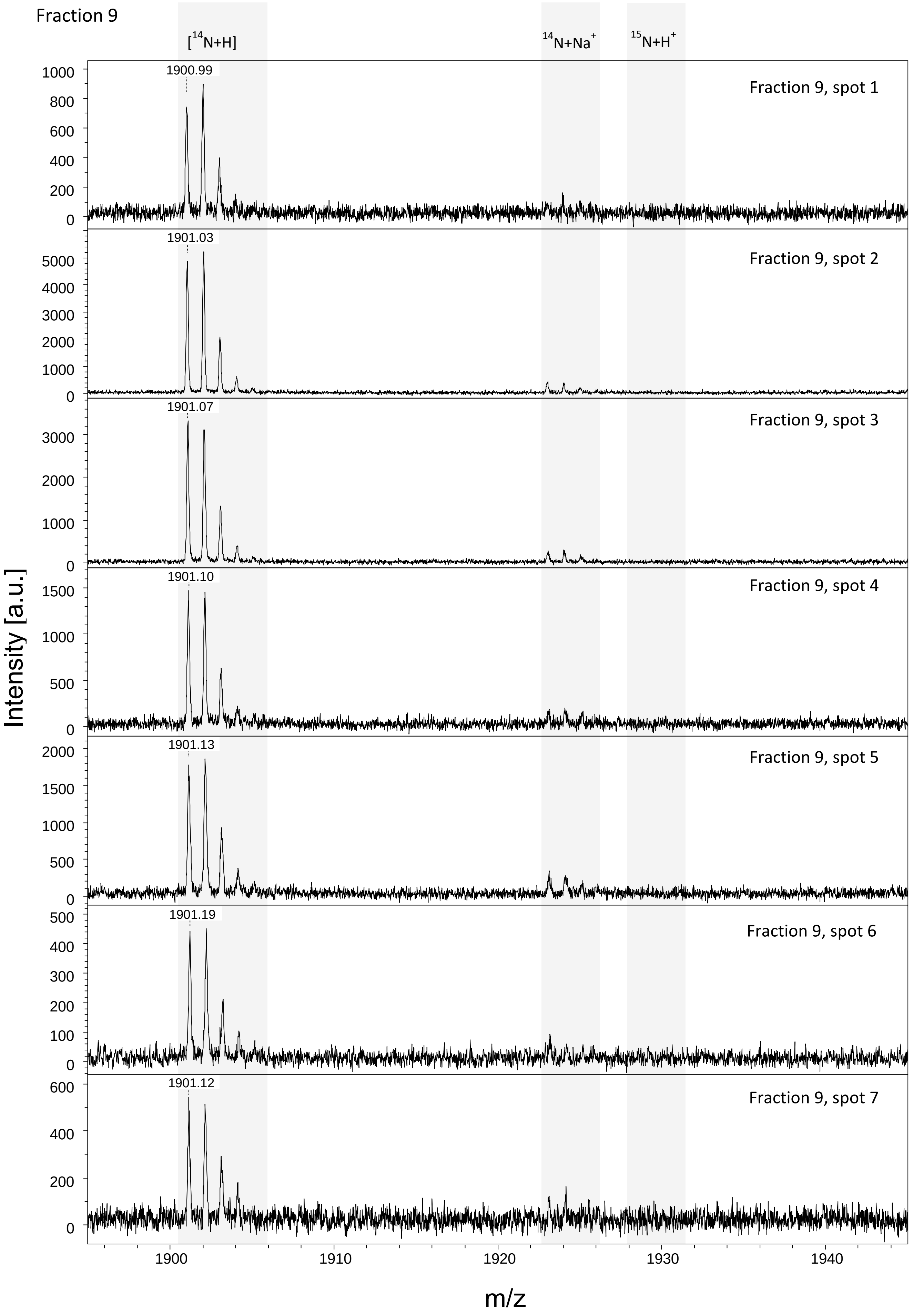
Fraction 9
[14N+H]
14N+Na+
15N+H+
1900.99
Fraction 9, spot 1
1901.03
Fraction 9, spot 2
1901.07
Fraction 9, spot 3
1901.10
Fraction 9, spot 4
1901.13
Fraction 9, spot 5
1901.19
Fraction 9, spot 6
1901.12
Fraction 9, spot 7
Intensity [a.u.]
m/z
1900
1910
1920
1930
1940

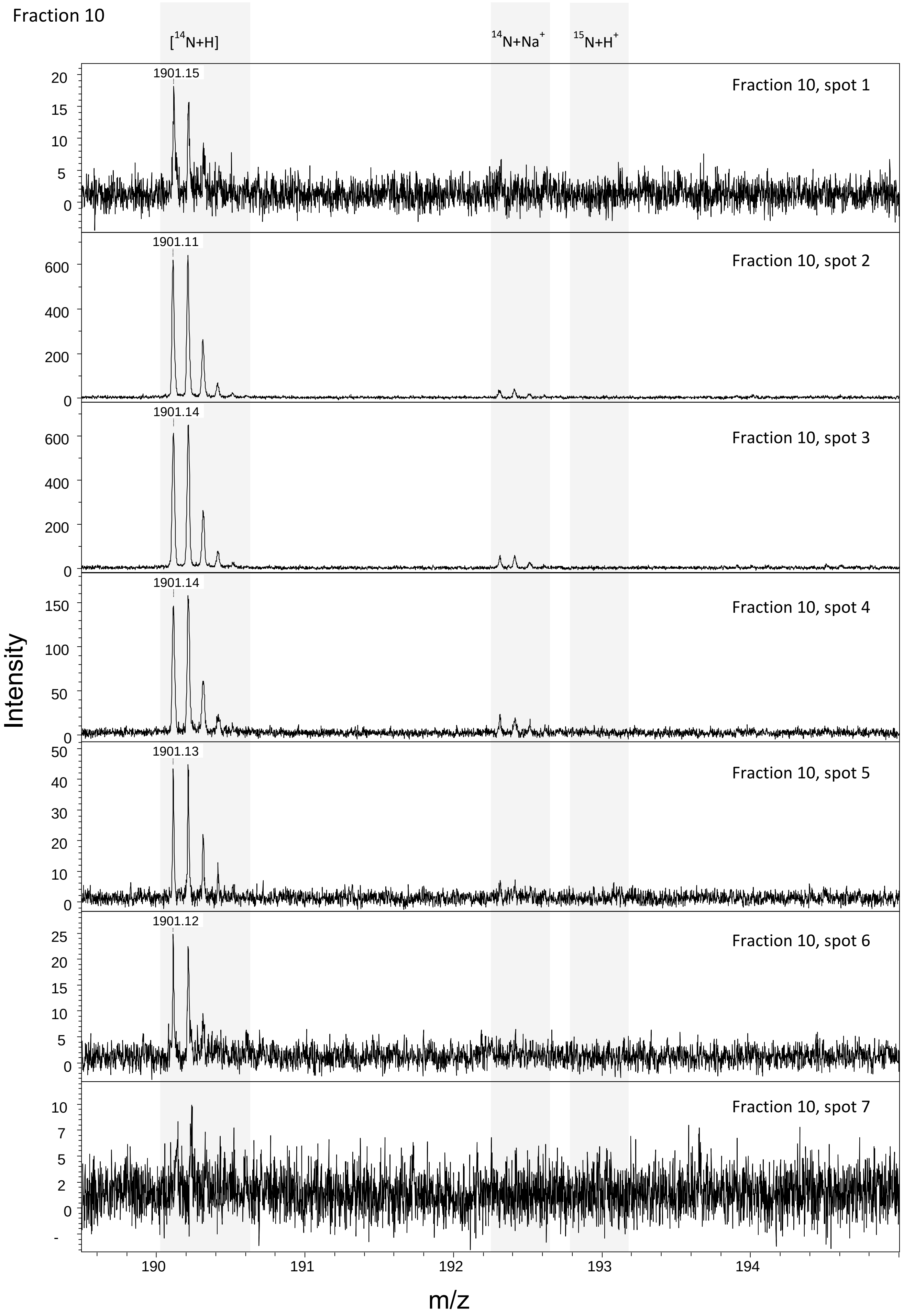
Fraction 10
[14N+H]
14N+Na+
15N+H+
1901.15
Fraction 10, spot 1
1901.11
Fraction 10, spot 2
1901.14
Fraction 10, spot 3
1901.14
Fraction 10, spot 4
1901.13
Fraction 10, spot 5
1901.12
Fraction 10, spot 6
Fraction 10, spot 7
Intensity
190
191
192
193
194
m/z

Figure S5. LC-MALDI-TOF-TOF spectra of all spots for oligomer fractions eluting between 8 and 9 ml (fraction 8), between 9 and 0 mL (fraction 9) and between 10 and 11 mL (fraction 10).

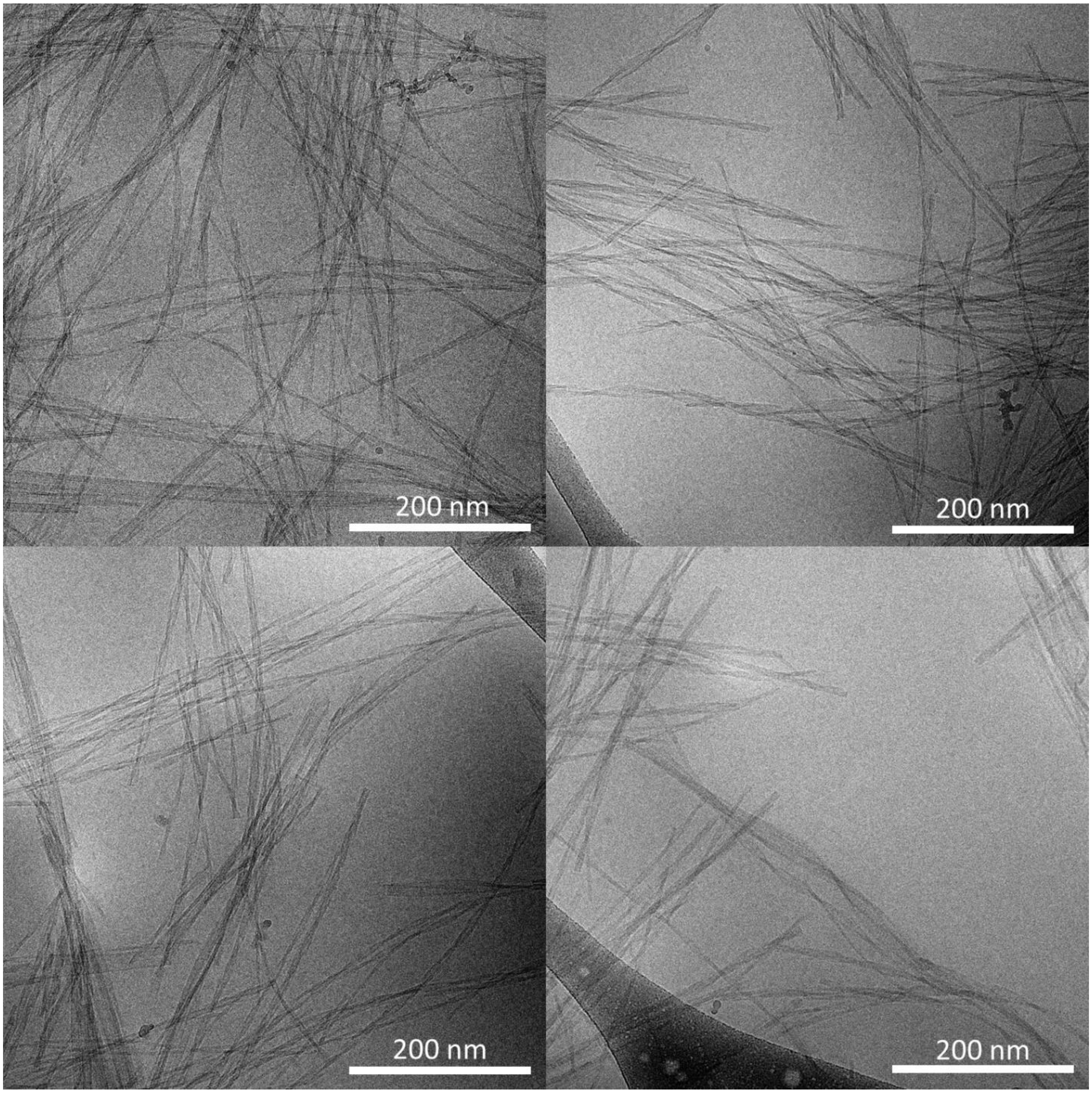

Figure S6. Additional frames of the cryo-EM imaging of tau fibrils at the plateau in X34 fluorescence.

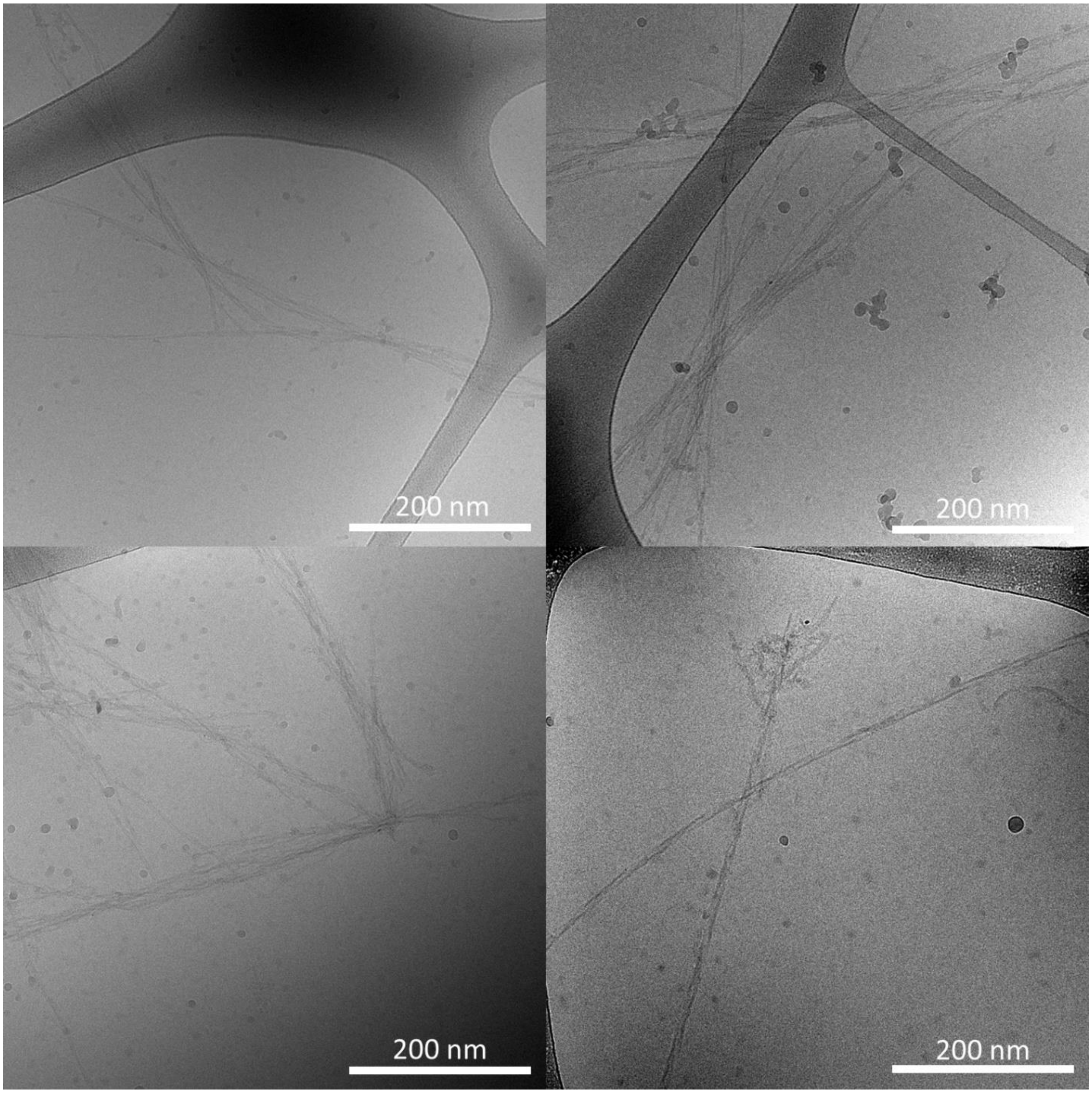

Figure S7. Additional frames of the cryo-EM imaging of tau fibrils at $t_{1/2}$ of the kinetics run.
